# Clinical and Biological Factors Are Associated with Treatment-Resistant Depression

**DOI:** 10.3390/bs12020034

**Published:** 2022-02-03

**Authors:** Massimiliano Buoli, Enrico Capuzzi, Alice Caldiroli, Alessandro Ceresa, Cecilia Maria Esposito, Cristina Posio, Anna Maria Auxilia, Martina Capellazzi, Ilaria Tagliabue, Teresa Surace, Francesca Legnani, Luisa Cirella, Martina Di Paolo, Guido Nosari, Francesco Zanelli Quarantini, Massimo Clerici, Fabrizia Colmegna, Antonios Dakanalis

**Affiliations:** 1 Department of Neurosciences and Mental Health, Fondazione IRCCS Ca’ Granda Ospedale Maggiore Policlinico, 20122 Milan, Italy; massimiliano.buoli@unimi.it (M.B.); cecilia.esposito@unimi.it (C.M.E.); criposio@gmail.com (C.P.); teresa.surace70@gmail.com (T.S.); francesca.legnani@unimi.it (F.L.); luisa.cirella@policlinico.mi.it (L.C.); martina.dipaolo@unimi.it (M.D.P.); guido.nosari@policlinico.mi.it (G.N.); quarantini24@gmail.com (F.Z.Q.); 2 Department of Pathophysiology and Transplantation, University of Milan, 20122 Milan, Italy; 3 Psychiatric Department, Azienda Socio-Sanitaria Territoriale Monza, 20900 Monza, Italy; e.capuzzi1@campus.unimib.it (E.C.); alyscaldi@gmail.com (A.C.); massimo.clerici@unimib.it (M.C.); f.colmegna@hsgerardo.org (F.C.); 4 Department of Medicine and Surgery, University of Milano Bicocca, 20900 Monza, Italy; a.auxilia@campus.unimib.it (A.M.A.); martina.capellazzi@gmail.com (M.C.); i.tagliabue5@campus.unimib.it (I.T.); antonios.dakanalis@unimib.it (A.D.)

**Keywords:** Major Depressive Disorder (MDD), treatment-resistant depression (TRD), clinical features, biochemical markers

## Abstract

Background: Treatment-resistant depression (TRD) is a debilitating condition associated with unmet clinical needs. Few studies have explored clinical characteristics and serum biomarkers associated with TRD. Aims: We investigated whether there were differences in clinical and biochemical variables between patients affected by TRD than those without. Methods: We recruited 343 patients (165 males and 178 females) consecutively hospitalized for MDD to the inpatient clinics affiliated to the Fondazione IRCCS Policlinico, Milan, Italy (*n* = 234), and ASST Monza, Italy (*n* = 109). Data were obtained through a screening of the clinical charts and blood analyses conducted during the hospitalization. Results: TRD versus non-TRD patients resulted to be older (*p* = 0.001), to have a longer duration of illness (*p* < 0.001), to be more currently treated with a psychiatric poly-therapy (*p* < 0.001), to have currently more severe depressive symptoms as showed by the Hamilton Depression Rating Scale (HAM-D) scores (*p* = 0.016), to have lower bilirubin plasma levels (*p* < 0.001). In addition, more lifetime suicide attempts (*p* = 0.035), more antidepressant treatments before the current episode (*p* < 0.001), and a lower neutrophil to lymphocyte ratio at borderline statistically significant level (*p* = 0.060) were all associated with the TRD group. Conclusion: We identified candidate biomarkers associated with TRD such as bilirubin plasma levels and NLR, to be confirmed by further studies. Moreover, TRD seems to be associated with unfavorable clinical factors such as a predisposition to suicidal behaviors. Future research should replicate these results to provide robust data in support of the identification of new targets of treatment and implementation of prevention strategies for TRD.

## 1. Introduction

Major Depressive Disorder (MDD) is a highly prevalent mental disorder worldwide. It is a complex and heterogeneous illness, causing individual suffering, important health care costs, loss of productivity and increased suicide risk [1]. Furthermore, up to 60% of individuals with MDD may show an incomplete response to their first antidepressant treatment, whilst approximately 30% of patients respond poorly to various antidepressant treatments, eventually falling into the condition of treatment-resistant depression (TRD) [2]. Even though variously defined, the most common TRD definition requires a non-response to two or more consecutive antidepressant medications in a single major depressive episode [3]. Available data indicate that TRD differs from MDD without treatment resistance in several aspects. Patients with TRD have higher rates of relapse and account therefore for a greater economic burden than those without TRD [4]. In addition, TRD represents an important risk factor for the occurrence of several medical comorbidities, and it is positively associated with decreased life expectancy as a result of both natural and unnatural causes [5]. A number of clinical variables including older age, comorbid personality and anxiety spectrum disorders, longer duration of illness, more frequent and recurrent depressive episodes, greater severity of depression, suicidal behavior, higher rate of hospitalizations and medical comorbidity (e.g., cardiovascular diseases) were all detected as possible predictors of TRD [6,7]. However, in light of specific sociodemographic and clinical correlates associated with TRD, some authors hypothesized that TRD patients may differ in biological characteristics from non-TRD ones [8]. In support of this speculation in the last years, different research groups have tried to better understand the pathophysiology of TRD, focusing on the identification of candidate biomarkers associated with this condition [9]. Particularly, many studies and meta-analyses reported that, on average, TRD patients have increased serum levels of pro-inflammatory molecules including interleukin-6 (IL-6), tumor necrosis factor (TNF) and C-reactive protein (CRP) [10,11]. Of note, patients affected by MDD seem to benefit less from antidepressant treatments in the presence of hyperactivity of the inflammatory system [12]. In this regard, the neutrophil–lymphocyte ratio (NLR) was suggested as a reproducible biomarker of the degree of systemic inflammation in patients with different medical conditions including psychiatric ones [13]. Higher values of NLR may be associated with a better response to some antidepressants [14]. Moreover, some authors reported that over-inflammation may hamper the effectiveness of antidepressants as a result of lipid peroxidation, impaired mitochondrial function, insulin resistance, oxidative damage to neuron membranes and DNA [15,16]. Indeed, some antioxidant agents, including serum levels of uric acid, albumin and high-density lipoprotein cholesterol (HDL-C) were found to be lower among depressed patients than controls, whilst some oxidative damage products, including malondialdehyde, were higher than in healthy subjects [17]. In addition, bilirubin, an endogenous antioxidant, may be impaired by excessive oxidative stress [18]. It is not surprising that lower total serum bilirubin levels were proposed as a risk factor of vulnerability for different neurological and psychiatric disorders, including MDD [19]. Similarly, in light of the association between obesity and inflammation, metabolic syndrome and related biomarkers (e.g., dysregulated cortisol release) were hypothesized to be related to TRD [20,21].

Despite the available literature, the overall picture of TRD still seems conflicting and fragmentary. Indeed, few studies evaluated concomitantly different clinical variables and laboratory markers, especially among patients with TRD compared to non-TRD ones [22]. Nevertheless, it is noteworthy that most findings, in term of biomarkers in pathophysiology of MDD/TRD, are inconsistent in light of different confounding factors such as sample characteristics, ongoing medications, medical comorbidities and definition of TRD [9]. Thus, in order to gain more information useful for clinical practice and identify any specific feature of TRD patients, this study had the aim to explore the possible roles of several clinical variables and biochemical markers on the occurrence of TRD in a sample of real-world inpatients affected by unipolar MDD.

Therefore, our main research questions were:

1. What is the prevalence of TRD in hospitalized patients with MDD?
2. Are there some clinical and biological variables for discriminating TRD individuals from depressed patients without this characteristic?
3. Is it possible to hypothesize some interactions between the different biological systems implicated in TRD?

Given the exploratory nature of the selected clinical and biomarker panel based on previous available literature [13,17,23], it was expected that TRD patients may differ from non-TRD patients for some of the investigated variables.

## 2. Materials and Methods

An overall sample of 343 patients with a diagnosis of MDD according to DSM (Diagnostic and Statistical Manual of Mental Disorders) criteria [24] was enrolled among patients admitted to the inpatient clinics affiliated to (1) the Fondazione IRCCS Policlinico, Milan, Italy (*n* = 234), and (2) ASST Monza, Italy (*n* = 109). The protocol of this study was approved by the local Ethical Committees (approval number 1789). Clinical and biological data were obtained through a screening of the clinical charts, interviews with patients and their relatives, and blood analyses conducted during the first day of hospitalization. Exclusion criteria were the following: (1) an age < 18 years, (2) peripartum as perinatal depression presents a different clinical presentation associated with specific biological abnormalities [25,26]; (3) pharmacological treatments that could significantly change mood (e.g., steroids); (4) medical comorbidities that could significantly affect mood or biochemical parameters (e.g., rheumatoid arthritis) [27]. In case of psychiatric comorbidity, MDD represented the disorder that affected the patients for a longer time or/and was responsible for more disability. TRD was defined as the presence of at least two prior treatment failures at adequate dose and duration [28]. This criterion was adopted to identify TRD patients in our sample.

The following variables were collected on the first day of hospitalization:

- clinical variables: age, age at onset, duration of untreated illness (months), duration of illness (months), duration of hospitalization (days), duration of last antidepressant therapy (months), number of antidepressants assumed before the current episode, the presence of a current depressive episode with mixed features, presence of lifetime psychotic symptoms, presence of lifetime history of substance misuse, presence of lifetime history of misuse of more than one substance, presence of family history of psychiatric disorders, presence of family history of substance misuse, current treatment with more than one psychotropic drug (including benzodiazepines), comorbidity with at least one psychiatric diagnosis, comorbidity with more than one psychiatric diagnosis, presence of lifetime suicidal attempts, number of lifetime suicidal attempts, comorbidity with hypothyroidism, comorbidity with diabetes, comorbidity with obesity, the body mass index (BMI), and scores of Global Assessment of Functioning (GAF), Young Mania Rating Scale (YMRS), Hamilton Depression Rating Scale 21 items (HAM-D), Montgomery–Asberg Depression Rating Scale (MADRS), Brief Psychiatric Rating Scale (BPRS), Hamilton Anxiety Rating Scale (HAM-A);
- biological parameters: number of red blood cells (10^12/L), mean corpuscular volume (MCV) (fL), hemoglobin (HB) (g/dL), number of white blood cells (WBC) (10^9/L), number of lymphocytes (10^9/L), neutrophil-to-lymphocyte ratio (NLR), number of platelets (10^9/L), mean platelet volume (MPV) (fL), C-reactive protein (CRP) (mg/dL), total plasmatic proteins (g/dL), albumin (g/dL), bilirubin (mg/dL), uric acid (mg/dL), cholesterol (mg/dL), glycaemia (fasting) (mg/dL), creatine phosphokinase (CPK) (U/L), high-density lipoproteins (HDL) (mg/dL), low-density lipoproteins (LDL) (mg/dL), triglicerydes (mg/dL) and thyroid-stimulating hormone (TSH) (mcU/mL).

Duration of untreated illness was considered as the time elapsing between first episode of MDD and the prescription of a proper pharmacological treatment (antidepressant or quetiapine) [29]. Obesity was defined as a BMI ≥ 30 [30].

With regard to rating scales, GAF measures patients’ global functioning with total scores ranging from 1 to 100 and higher scores indicating less disability [31]. YMRS is a tool used to assess the severity of manic symptoms, and in the case of patients affected by MDD it is useful to identify mixed features (YMRS scores ≥ 10) [32]. Both HAM-D and MADRS measure the severity of depressive symptoms with the first focusing much more on anxiety-somatization aspects (HAM-D ≥ 8 indicating clinically significant depressive symptoms) [33] and the latter more specific to identify core symptoms of MDD (e.g., guilt feelings and anhedonia). MADRS scores ≥ 10 reveal clinically significant depressive symptoms [34]. BPRS assesses the severity of general psychopathology with a score ≥ 31 indicating the presence of clinically significant psychiatric symptoms [35] and, finally, HAM-A measures the severity of anxiety symptoms with a score ≥ 8 requiring clinical attention [36].

Given that a difference of at least 5 points in age is expected between TRD and non-TRD patients according to previous literature [37] and that a *p* value = 0.05 is considered statistically significant, for a power of 80% a sample of 212 subjects with at least 106 individuals for each subgroup is calculated to be reliable.

Statistical analyses were performed through The Statistical Package for Social Sciences (SPSS) for Windows (version 26.0). Descriptive analyses of the total sample were initially performed, then groups identified according to the presence of TRD were compared using Student’s *t*-tests for means of quantitative variables and Chi-square tests for qualitative variables (Table 1—clinical variables and Table 2—biological variables). Owing to the large number of factors statistically related to the dependent variable (the presence of TRD) at the univariate analyses, two multiple logistic regression analyses (one for clinical variables—Table 3 and one for biological ones—Table 4) were performed including only the statistically significant variables. This approach was followed as recommended by previous publications on purposeful selection of variables in logistic regression [38] and similarly to the method used by our group in other articles for the analyses of data from samples of patients affected by mood disorders [39]. The choice to initially perform two separate models for clinical and biological variables was driven by the hypothesis that no inter-relation was present between these two sets of factors [40]. A final model entering both clinical and biological variables was then performed to verify the stability of the results of the previous models [40] (Table 5). Finally, correlation analyses (Pearson’s r) were performed to detect eventual interactions among all the independent variables inserted in the final logistic regression model and explaining eventual differences in the findings of separate versus final regression models (Table 3 and Table 4 versus Table 5).

The goodness of fit of the models was assessed by the Omnibus and Hosmer–Lemeshow tests. The level of statistical significance was set at *p* ≤ 0.05, and confidence intervals at 95% for odds ratios were calculated.

## 3. Results

The total sample included 343 patients, of whom 165 were males (48.1%) and 178 females (52.1%). The mean age was 49.27 ± 14.98 years, with a minimum of 18 and a maximum of 86. Descriptive analyses and *p* values of univariate analyses are reported in Table 1 (demographic and clinical variables) and Table 2 (biological variables). With regard to antidepressant therapy assumed during the last depressive episode, 17 patients were treated with tricyclic antidepressants (TCAs), 69 with selective serotonin reuptake inhibitors (SSRIs), 42 with serotonin-norepinephrine reuptake inhibitors (SNRIs), 12 with mirtazapine, 12 with trazodone, 10 with vortioxetine, 7 with bupropion and 13 with other antidepressants. Patients without TRD were more frequently treated with SSRIs than TRD ones (36.8% versus 19.3%, *p* < 0.05).

First of all, considering demographical and clinical variables, there were not significant differences between TRD and non-TRD patients (*p* > 0.05) in: age at onset, duration of untreated illness, duration of hospitalization, duration of last antidepressant therapy, presence of mixed features during the current episode, presence of lifetime psychotic symptoms, presence of lifetime history of substance misuse, presence of lifetime history of more than one substance, family history of psychiatric disorders, family history of substance abuse, frequency of current treatment with statins, comorbidity with at least one psychiatric diagnosis, comorbidity with more than one psychiatric diagnosis, history of lifetime suicidal attempts, comorbidity with hypothyroidism, comorbidity with obesity, BMI, comorbidity with diabetes, GAF score, YMRS score, MADRS score, BPRS score, HAM-A score. In contrast, TRD versus non-TRD patients were older (*p* = 0.001), and they showed: a longer duration of illness (*p* < 0.001), a higher number of antidepressants assumed before the current episode (*p* < 0.001), a higher frequency of a current therapy with more than one psychotropic drug (*p* = 0.001), more lifetime suicidal attempts (*p* = 0.019) and higher HAM-D scores at the moment of hospitalization (*p* = 0.016).

With regards to biological parameters, there were no differences between the two groups (*p* > 0.05) in: number of red blood cells (RBC), hemoglobin, number of white blood cells (WBC), number of lymphocytes, number of platelets, mean platelet volume (MPV), C-reactive protein (CRP), total plasmatic proteins, albumin, uric acid, total cholesterol, HDL LDL, triglycerides, glycaemia (fasting), CPK, TSH. In contrast, TRD versus non-TRD patients showed lower NLR (*p* = 0.012) and bilirubin (*p* < 0.001) levels and a smaller MCV at a borderline statistically significant level (*p* = 0.069).

With regard to the binary logistic regression with clinical variables (Table 3), the goodness-of-fit test (Hosmer and Lemeshow Test: χ^2^ = 6.773, *p* = 0.561) showed that the model was reliable, allowing for a correct classification of 83.1% of the cases. In addition, the model was overall significant (Omnibus test: χ^2^ = 68.612, df = 5, *p* < 0.001). TRD patients (versus non-TRD ones) were confirmed to have received more antidepressants before the current episode (*p* < 0.001) and to show more lifetime suicidal attempts (*p* = 0.035).

With regard to the binary logistic regression with biological variables including MCV in light of the borderline statistically significant difference between groups (Table 4), the goodness-of-fit test (Hosmer and Lemeshow Test: χ^2^ = 7.440, *p* = 0.490) showed that the model was reliable, allowing for a correct classification of 66.7% of the cases. In addition, the model was overall significant (Omnibus test: χ^2^ = 14.585, df = 3, *p* = 0.002). A lower NLR was confirmed to be associated with TRD patients at the borderline statistically significant level (*p* = 0.060).

The final model (Table 5) confirmed substantially the results of the previous model with the exception of a loss of statistical strength about the association between NLR and TRD and a more robust association between more lifetime suicide attempts and TRD. Duration of illness reached statistical significance, but with an opposite trend with respect to univariate analyses. Of note, correlation analyses revealed a significant inverse interaction between bilirubin levels and number of suicide attempts (r = −0.189, *p* < 0.01) and between duration of illness and NLR (r = −0.195, *p* < 0.01). In addition, NLR and bilirubin resulted to have a direct correlation (r = 0.233, *p* < 0.01).

## 4. Discussion

Our study focused on the entanglement of clinical variables and biochemical parameters in the occurrence of TRD in quite a large sample of patients hospitalized for MDD. In line with literature [37,41], TRD was present in slightly more than one-third (35.9%) of the recruited sample. In addition, with regard to rating scales, our sample showed scores that are reliable for inpatients with a diagnosis of MDD (HAM-D and MADRS mean total scores with standard deviations corresponding to a moderate-to-severe major depressive episode) [34,42].

Either clinical or biological differences emerged between patients suffering from TRD and non-TRD ones. First of all, it is not surprising that patients without TRD were more frequently treated with SSRIs than TRD ones (36.8% versus 19.3%). Indeed, the majority of the clinical practice guidelines recommend SSRI as first-line treatment thanks to their favourable risk–benefit ratio [43]. On the other hand, SNRIs and TCAs are commonly considered as a second-line option or for patients with more severe depressive symptoms [44]. Specifically, TCAs have a less favourable safety profile with respect to SSRIs and SNRIs, and they are more associated with troublesome side effects (e.g., constipation) that prevent an optimal adherence to pharmacological treatment [45].

With regard to clinical variables, TRD patients showed features typically associated with less clinical stabilization and overall more severity of MDD. Indeed, albeit revealed only by the univariate analyses, TRD versus non-TRD patients had a significantly longer duration of illness and a higher frequency of a current therapy with more than one psychotropic drug. The prescription of a polytherapy and a chronic course of illness are recognized factors associated with poor response to pharmacological treatment in MDD [37,46,47]. Specifically, patients with TRD are often treated with combined treatments (e.g., antidepressants plus atypical antipsychotics or lithium) associated with an increased risk of side effects and less adherence to medical prescription, resulting in a further worsening of symptoms [48]. This is consistent with our result, in line with previous literature, that patients with TRD experience more different antidepressant treatments with respect to non-TRD ones [49,50]. Furthermore, in our sample, a more severe suicidal behaviour resulted to be robustly associated with TRD, as showed by the binary logistic regression models. Several authors underlined the association between suicidal behaviour and poor response to treatment in MDD, suggesting that history of self-harm [50,51,52] or number of suicidal attempts are strong predictors of TRD [53,54,55].

In terms of biological parameters, even though not confirmed by logistic regression, TRD versus non-TRD patients seem to have lower bilirubin levels. Lower bilirubin levels were also found to be correlated with more lifetime suicide attempts, thus confirming that antioxidant defenses can be compromised in severe patients. Based on the authors’ knowledge, this is the first study reporting a potential role between bilirubin levels and MDD severity. Bilirubin has antioxidant and anti-inflammatory properties, and it is assumed that increased oxidative stress might impair its functioning [19]. Although in recent times a growing number of studies have explored the involvement of anti-oxidative defences in the pathophysiology of MDD [56,57,58], there is little evidence concerning the role played by bilirubin, suggesting that lower levels might represent a risk to more severe forms of illness [19,59]. Of note, some authors proposed bilirubin as a promising therapy for the management of neurodegenerative diseases like Parkinson’s Disease [60]. Similarly, a lower NLR resulted to be associated with TRD. NLR is commonly considered as a reliable measure of subclinical inflammation [61], as well as a potential biomarker associated to the severity of MDD and propensity to suicidal behaviour [62,63]. In contrast with our findings, most of the literature data highlighted higher NLR levels in depressed patients compared to healthy controls [64,65]. Similarly, other studies reported higher NLR in suicide attempters than individuals without suicidal behaviour [63,66]. Conversely, there is evidence that antidepressants treatment might influence inflammatory processes by affecting proliferation of lymphocyte, hence decreasing NRL [67,68]. In addition, the finding of a lower NLR in TRD versus non-TRD patients is consistent with data reported by our group on a different sample showing that lower NLR levels are associated with violent suicide attempts [13]. Even though we did not focus on the modalities of self-harm, TRD patients of this sample resulted to have more lifetime suicidal attempts, consistently with the result of a lower NLR with respect to patients without TRD.

Some limitations should be considered in the interpretation of the aforementioned results. First, even though the sample was quite large, the recruitment in only two centers might partly affect the generalization of our results. Second, last pharmacological treatment with different compounds could have influenced either clinical or biological parameters, as non-TRD patients were more often treated with SSRI. Third, the information partly obtained interviewing patients and their relatives (e.g., family history of psychiatric disorders) may not be always accurate. Fourth, with regard to some variables, a lot of data are missing either because this information is not routinely collected in one of the two inpatient clinics or because it cannot be derived from medical records. Multiple imputation was not performed in light of the retrospective naturalistic design of our study.

From a clinical point of view, our data indicate that patients with TRD should be managed considering the increased risk of suicide and a probable weakness of antioxidant systems compared to non-TRD patients. With regard to suicidal risk, clinicians may consider pharmacological approaches, prescribing compounds with a clear anti-suicidal effect (e.g., lithium) [69], psychosocial interventions (e.g., psychoeducation) [70] or neuromodulation techniques (e.g., vagus nerve stimulation) [71]. However, it is necessary to underline that a recent review highlighted that these strategies showed poor efficacy in samples of patients with TRD [53]. Studies assessing the effectiveness of different treatments to mitigate self-harm risk in TRD patients should be therefore absolutely promoted for the best management of subjects affected by this condition. In this context, as regards the enhancement of antioxidative systems, it would be important to understand whether the administration of antioxidant agents such as vitamin E [72] could improve some clinical aspects of patients with TRD.

In light of the previous considerations, the results of the present study corroborate the hypothesis that TRD patients differ from non-TRD ones for some clinical and biological variables, thus potentially providing insight into the pathophysiology and treatment of this severe condition. Future research should replicate these results to provide robust data in support of the identification of new targets of treatment and implementation of prevention strategies for TRD. Furthermore, these data stimulate a greater deepening of the relationships between the different clinical and biological markers in explaining the onset and course of mood disorders as well as the search for treatments that can promote antioxidative defenses in the most serious patients. These research directions will favor an approach to the patients with TRD based on precision medicine.

## Figures and Tables

**Table 1:** Clinical variables of the total sample and of the two groups divided according to the presence of TRD.

Variables	Total Sample *n* = 343	Non-TRD *n* = 220 (64.1%)	TRD *n* = 123 (35.9%)	*p* Value
Age (years)	49.27 (±14.98)	47.20 (±15.34)	52.99 (±13.60)	**0.001**
Age at onset of MDD (years) (Missing = 35)	37.31 (±16.41)	37.61 (±16.78)	36.79 (±15.79)	0.677
Duration of untreated illness (months) (Missing = 125)	52.69 (±104.44)	48.05 (±102.84)	60.69 (±107.33)	0.390
Duration of illness (months) (Missing = 38)	138.01 (±157.88)	107.90 (±140.99)	190.71 (±172.08)	**<0.001**
Duration of hospitalization (days) (Missing = 113)	12.90 (±9.08)	12.98 (±8.63)	12.75 (±9.85)	0.856
Duration of last antidepressant therapy (months) (Missing = 168)	9.61 (±19.58)	11.23 (±21.56)	7.12 (±15.88)	0.149
Number of antidepressants assumed before the current episode (Missing = 154)	1.32 (±1.76)	0.68 (±0.85)	2.71 (±2.37)	**<0.001**
Current depressive episode with mixed features (Missing = 114)	36 (15.7%)	23 (15.9%)	13 (15.5%)	1.000
Presence of lifetime psychotic symptoms (Missing = 116)	51 (22.5%)	28 (19.4%)	23 (27.7%)	0.186
Presence of lifetime history of substance misuse (Missing = 7)	89 (26.5%)	57 (26.3%)	32 (26.9%)	1.000
Presence of lifetime history of misuse of more than one substance (Missing = 7)	23 (6.8%)	17 (7.8%)	6 (5.0%)	0.376
Presence of family history of psychiatric disorders (Missing = 140)	92 (45.3%)	54 (41.9%)	38 (51.4%)	0.241
Presence of family history of substance misuse (Missing = 149)	16 (8.2%)	10 (7.9%)	6 (8.8%)	1.000
Current therapy with more than one psychotropic drug (Missing = 116)	177 (78.0%)	102 (70.8%)	75 (90.4%)	**0.001**
Current treatment with statins (Missing = 222)	9 (7.4%)	3 (4.2%)	6 (12.0%)	0.160
Comorbidity with at least one psychiatric diagnosis (Missing = 115)	78 (34.2%)	46 (32.4%)	32 (37.2%)	0.474
Comorbidity with more than one psychiatric diagnosis (Missing = 113)	9 (3.9%)	6 (4.2%)	3 (3.5%)	1.000
Presence of lifetime suicidal attempts (Missing = 66)	128 (46.2%)	72 (42.6%)	56 (51.9%)	0.140
Number of lifetime suicidal attempts (Missing = 69)	0.73 (±1.02)	0.61 (±0.88)	0.92 (±1.19)	**0.019**
Comorbidity with hypothyroidism (Missing = 113)	14 (6.1%)	6 (4.2%)	8 (9.3%)	0.154
Comorbidity with obesity (Missing = 246)	26 (26.8%)	10 (19.6%)	16 (34.8%)	0.111
BMI (Missing = 261)	23.44 (±3.07)	23.21 (±3.11)	23.80 (±3.03)	0.399
Comorbidity with diabetes (Missing = 115)	15 (6.6%)	6 (4.2%)	9 (10.5%)	0.096
GAF score (Missing = 116)	46.48 (±15.30)	47.33 (±15.50)	45.06 (±14.93)	0.280
YMRS score (Missing = 122)	3.59 (±4.75)	3.61 (±4.75)	3.55 (±4.79)	0.925
HAM-D score (Missing = 124)	15.95 (±5.57)	15.25 (±5.28)	17.12 (±5.86)	**0.016**
MADRS score (Missing = 128)	24.87 (±6.77)	24.50 (±6.87)	25.53 (±6.59)	0.285
BPRS score (Missing = 104)	36.75 (±7.82)	36.47 (±7.83)	37.21 (±7.84)	0.481
HAM-A score (Missing = 133)	8.98 (±4.75)	8.61 (±4.38)	9.62 (±5.31)	0.137

BMI = Body Mass Index; BPRS = Brief Psychiatric Rating Scale; GAF = Global Assessment of Functioning; HAM-A = Hamilton Rating Scale for Anxiety; HAM-D = Hamilton Rating Scale for Depression; MADRS = Montgomery–Asberg Depression Rating Scale; MDD = Major Depressive Disorder; TRD = Treatment-Resistant Depression; YMRS = Young Mania Rating Scale. Note: For some variables, more than 100 values are missing as they are not routinely collected or are not available in one of the involved inpatient clinics. Means for quantitative variables and frequencies for qualitative ones are reported. Standard deviations for quantitative variables and percentages for qualitative variables are reported in brackets. In bold, statistically significant *p* resulting from χ^2^ or unpaired Student’s *t*-tests.

**Table 2:** Biological markers in study sample stratified by TRD.

Variables	Total Sample *n* = 343	Non-TRD *n* = 220 (64.1%)	TRD *n* = 123 (35.9%)	*p* Value
Number of RBC (10^12/L) (Missing = 44)	4.65 (±0.52)	4.61 (±0.53)	4.65 (±0.51)	0.494
MCV (fL) (Missing = 153)	86.63 (±6.47)	87.29 (±5.94)	85.53 (±7.18)	0.069
HB (g/dL) (Missing = 44)	13.73 (±1.72)	13.81 (±1.65)	13.58 (±1.82)	0.281
Number of WBC (10^9/L) (Missing = 45)	7.55 (±2.72)	7.67 (±2.94)	7.34 (±2.30)	0.321
Number of RBC (10^12/L) (Missing = 44)	4.65 (±0.52)	4.61 (±0.53)	4.65 (±0.51)	0.494
Number of lymphocytes (10^9/L) (Missing = 115)	2.30 (±0.83)	2.24 (±0.85)	2.39 (±0.79)	0.187
NLR (Missing = 114)	2.23 (±1.86)	2.46 (±2.14)	1.89 (±1.26)	**0.012**
Number of PLT (10^9/L) (Missing = 150)	243.90 (±69.00)	245.13 (±73.65)	241.83 (±60.82)	0.749
MPV (fL) (Missing = 157)	12.11 (±17.88)	12.89 (±22.52)	10.78 (±1.08)	0.438
CRP (mg/dL) (Missing = 250)	1.37 (±2.77)	1.51 (±3.33)	1.16 (±1.69)	0.551
Total plasmatic proteins (g/dL) (Missing = 163)	6.59 (±0.56)	6.59 (±0.61)	6.60 (±0.48)	0.925
Albumin (g/dL) (Missing = 150)	4.19 (±0.40)	4.18 (±0.43)	4.19 (±0.35)	0.872
Bilirubin (mg/dL) (Missing = 63)	0.62 (±0.53)	0.70 (±0.60)	0.49 (±0.36)	**<0.001**
Uric acid (mg/dL) (Missing = 159)	4.61 (±1.36)	4.62 (±1.44)	4.60 (±1.26)	0.934
Cholesterol (mg/dL) (Missing = 84)	185.06 (±42.61)	181.91 (±42.27)	190.14 (±42.87)	0.131
HDL (mg/dL) (Missing = 220)	52.41 (±18.59)	52.58 (±21.00)	52.11 (±13.86)	0.891
LDL (mg/dL) (Missing = 230)	112.97 (±36.04)	115.75 (±36.07)	108.10 (±35.91)	0.280
Triglicerydes (mg/dL) (Missing = 216)	115.77 (±63.10)	113.38 (±63.40)	119.57 (±63.09)	0.593
Glycaemia (fasting) (mg/dL) (Missing = 53)	92.03 (±19.82)	91.78 (±17.97)	92.48 (±22.77)	0.771
CPK (U/L) (Missing = 169)	116.40 (±165.57)	125.71 (±174.60)	102.90 (±151.70)	0.373
TSH (mcU/mL) (Missing = 126)	1.93 (±1.73)	1.97 (±1.99)	1.87 (±1.20)	0.670

CPK = creatine phosphokinase; CRP = C-reactive protein; HB = haemoglobin; RBC = red blood cells; MCV = mean corpuscular volume; MPV = mean platelet volume; NLR = neutrophil-to-lymphocyte ratio; PLT = platelets; TRD = Treatment-Resistant Depression; TSH = thyroid-stimulating hormone; WBC = white blood cells. Note: For some variables, more than 100 values are missing as they are not routinely collected or are not available in one of the involved inpatient clinics. Means and standard deviations (in brackets) are reported. In bold, statistically significant *p* resulting from unpaired Student’s *t*-tests.

**Table 3:** Summary of the statistics of best-fit binary regression model for quantitative clinical variables.

Variables	B	S.E.	Wald	*p*	OR	95% CI for OR
Age (years)	0.025	0.013	3.509	0.061	1.025	0.999–1.052
Duration of illness (months) (Missing = 38)	−0.001	0.002	0.772	0.380	0.999	0.996–1.002
Number of antidepressants assumed before the current episode (Missing = 154)	1.110	0.211	27.624	**<0.001**	3.034	2.006–4.589
Number of lifetime suicidal attempts (Missing = 69)	0.411	0.195	4.431	**0.035**	1.509	1.029–2.213
HAM-D score (Missing = 124)	0.069	0.039	3.163	0.075	1.071	0.993–1.156

In this analysis, the dependent variable was presence of Treatment-Resistant Depression (TRD) versus absence of TRD. B = regression coefficient; CI = confidence interval; HAM-D = Hamilton Rating Scale for Depression; OR = odds ratio; S.E. = standard error of B; Wald = Wald statistics. In bold, statistically significant *p* resulting from the model.

**Table 4:** Summary of the statistics of best-fit binary regression model for quantitative biological variables.

Variables	B	S.E.	Wald	*p*	OR	95% CI for OR
NLR (Missing = 114)	−0.235	0.125	3.529	0.060	0.790	0.618–1.010
Bilirubin (mg/dL) (Missing = 63)	−0.695	0.411	2.852	0.091	0.499	0.223–1.118
MCV (fL) (Missing = 153)	−0.039	0.025	2.496	0.114	0.962	0.917–1.009

In this analysis, the dependent variable was presence of Treatment Resistant Depression (TRD) versus absence of TRD. B = regression coefficient; CI = confidence interval; MCV = mean corpuscular volume; NLR = neutrophil to lymphocyte ratio; OR = odds ratio; S.E. = standard error of B; Wald = Wald statistics. In bold, statistically significant *p* resulting from the model.

**Table 5:** Regression analysis predictors of TRD.

Variables	B	S.E.	Wald	*p*	OR	95% CI for OR
Age (years)	0.035	0.020	3.233	0.072	1.036	0.997–1.076
Duration of illness (months) (Missing = 38)	−0.005	0.002	4.213	**0.040**	0.995	0.991–1.000
Number of antidepressants assumed before the current episode (Missing = 154)	1.485	0.318	27.791	**<0.001**	4.416	2.367–8.238
Number of lifetime suicidal attempts (Missing = 69)	0.662	0.248	7.112	**0.008**	1.939	1.192–3.154
HAM-D score (Missing = 124)	0.110	0.057	3.698	0.054	1.16	0.998–1.248
NLR (Missing = 114)	−0.294	0.192	2.348	0.125	0.512	0.618–1.085
Bilirubin (mg/dL) (Missing = 63)	−0.337	0.464	0.529	0.467	0.714	0.287–1.772
MCV (fL) (Missing = 153)	−0.076	0.057	1.777	0.183	0.927	0.828–1.037

In this analysis, the dependent variable was presence of Treatment Resistant Depression (TRD) versus absence of TRD. B = regression coefficient; CI = confidence interval; HAM-D = Hamilton Rating Scale for Depression; MCV = mean corpuscular volume; NLR = neutrophil to lymphocyte ratio; OR = odds ratio; S.E. = standard error of B; Wald = Wald statistics. In bold, statistically significant *p* resulting from the model.

## Data Availability

The data presented in this study are available upon request to the corresponding author.
